# Severe B12 Deficiency Causing a Maturation Defect Mimicking Myelodysplastic Syndrome With Excess Blasts

**DOI:** 10.7759/cureus.60837

**Published:** 2024-05-22

**Authors:** Benjamin Mecham, Wassim Drissi, Gage Brummell, Neelakanta Dadi, David E Martin

**Affiliations:** 1 Graduate Medical Education, Unity Health, Searcy, USA; 2 Hematology/Oncology, Unity Health, Searcy, USA

**Keywords:** severe anemia, thrombocytopenia, severe neutropenia, megaloblastic, hypercellular bone marrow, myelodysplastic syndrome mimic, bone marrow biopsy, vit b12 deficiency, myelodysplastic syndrome (mds), “pancytopenia”

## Abstract

Vitamin B12 deficiency is a common condition that is often asymptomatic, though in severe cases may cause megaloblastic anemia and even neurologic symptoms. Occasionally, the clinical presentation can include pancytopenia and thus mimic a more concerning myelodysplastic syndrome (MDS) until corrected by B12 supplementation. In this unusual case, we present a patient with B12 deficiency who presents with severe macrocytic anemia, neutropenia, lymphocytosis, and a bone marrow morphology consistent with MDS.

## Introduction

Vitamin B12 deficiency is a common condition estimated to be present in 3.6% of all adults over the age of 18 years that often presents with fatigue caused by macrocytic anemia [1]. However, it can have diverse clinical manifestations ranging from asymptomatic to severe neurological and hematological abnormalities that lead to death if untreated [2]. Despite its prevalence, diagnosing B12 deficiency can be challenging due to its varied presentation, particularly when it mimics other hematological disorders like myelodysplastic syndrome (MDS) [3-5]. Vitamin B12 deficiency can mimic MDS due to its effects on the bone marrow, causing stunted hematopoiesis leading to the production of large, abnormally nucleated red blood cells [3-5]. In B12 deficiency, the bone marrow produces megaloblasts that resemble the dysplastic cells, as well as cause leukopenia and thrombocytopenia, all seen with MDS [3-5]. Unlike MDS, which requires treatment with hypomethylating agents, the consequences of B12 deficiency can be harmlessly reversed with simple supplementation [3-7]. This emphasizes the importance of making the correct diagnosis despite an atypical presentation, which the following case illustrates. We present the case of a patient who presents with severe macrocytic anemia, neutropenia, lymphocytosis, and a bone marrow morphology consistent with MDS.

## Case presentation

The patient is a 76-year-old female who was admitted to the US hospital with severe pancytopenia found at her primary care physician's (PCP) office. She had been complaining of fatigue and not feeling well for about a month prior. Her physical exam was unremarkable. At the hospital, her initial labs included hemoglobin (Hgb) of 4.3 g/dL, hematocrit of 12.7%, mean corpuscular volume (MCV) of 130 fL, mean corpuscular hemoglobin (MCH) of 45.3 pg, platelet count of 80,000/µL, and white blood cell count (WBC) of 1900/µL. The differential showed severe neutropenia and relative lymphocytosis. She received three units of blood transfusion during the hospital stay. A peripheral blood smear was reviewed and showed tear drop cells, no schistocytes or blasts, dysplastic white blood cells, and normal number and appearance of platelets. This included hypersegmented neutrophils, consistent with either vitamin B12 deficiency or MDS. Flow cytometry was negative for acute leukemia/lymphoproliferative disorder. On day two of admission, her iron levels were elevated, and folate was normal, but she was found to be severely B12 deficient with a level of 27 pg/mL. Supplementation was started with weekly intramuscular 1000 µg vitamin B12 shots. On hospital day four, the patient had a bone marrow biopsy and was discharged the following day. At the time of discharge from the hospital, she remained leukopenic with a WBC of 2900/µL, had a stable Hgb of 9.2 g/dL, and a decreased platelet count of 38,000/µL. She did receive one unit of transfused platelets before discharge. 

The patient was seen four days after discharge in the hematology clinic. The patient reported receiving B12 shots at home and that her breathing and fatigue had significantly improved after the blood transfusions. A verbal report from pathology was that the bone marrow biopsy showed dysplastic changes consistent with MDS with about seven percent blasts. Patient labs included WBC of 3220/µL, Hgb of 10.3 g/dL, and normal platelet count of 255,000/µL.

**Figure 1:**
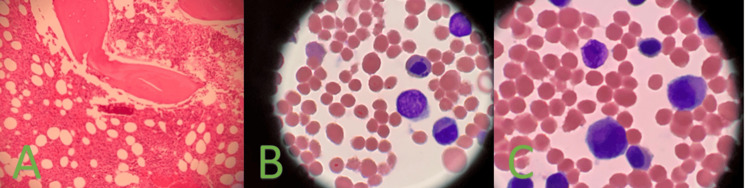
(A) 20x hematoxylin and eosin-stained bone marrow core showing hypercellularity, (B) 100X Giemsa-stained aspirate showing megaloblastic changes, (C) 100X Giemsa-stained aspirate showing irregular erythroid nucleus and megaloblastic changes in all lineages

One month after discharge the patient's labs improved with WBC of 5000/µL, Hgb of 10.9 g/dL, and platelet count of 226,000/µL. The bone marrow biopsy report showed 65% cellularity with a left shift in myeloid maturation, increased erythrocyte precursors, and decreased megakaryocytes. Hypercellular marrow showing trilineage hematopoiesis with erythroid hyperplasia and a myeloid left shift with increased blasts. Blasts comprise approximately 7% of total marrow cells by visual exam. MDS extended fluorescence in situ hybridization (FISH) was negative. The reported diagnosis was MDS with excess blasts and decreased iron stores by Prussian blue stain. Later, the diagnosis was changed by addendum to profound marrow changes accompanying vitamin B12 deficiency (Figure 1).

**Figure 2:**
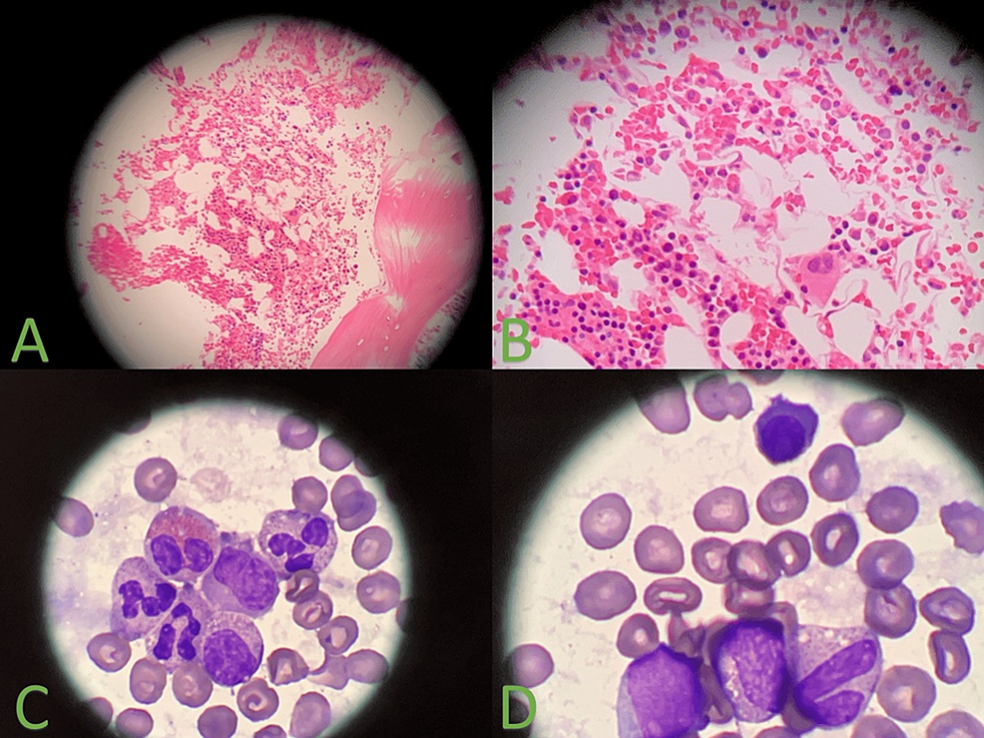
(A) 20x hematoxylin and eosin-stained bone marrow core showing normocellularity, (B) 40X hematoxylin and eosin-stained bone marrow showing that marrow abnormalities are resolved, (C,D) 100X Giemsa-stained aspirate showing normal morphology

Given the patient's improvement with B12 shots at home, a repeat bone biopsy was performed and followed up at the three-month mark. The patient's labs showed near normal counts: WBC, 7000/µL; Hgb, 12.6 g/dL; MCV, 98; platelet count, 276,000/µL. The repeat bone marrow biopsy showed normocellular bone marrow and no evidence of any leukemia or lymphoma (Figure 2). This is reflective of a vitamin B12 deficiency. The patient declined any further workup to evaluate for pernicious anemia but will continue with oral vitamin B12 supplementation.

## Discussion

Vitamin B12 deficiency is normally a harmless condition that is easily recognized and treated with supplementation. Although many diseases and conditions have typical presentations, this case is an example commonly seen in medical issues with simple treatments that can present in bizarre ways, even mimicking other diseases. MDS is a concerning diagnosis to find on bone marrow biopsy, with progression to acute myeloid leukemia having a potentially poor prognosis [8]. This case underlines the significance of understanding the pathophysiology of the disease, such as the role of vitamin B12 in hematopoiesis, which is vital in understanding how MDS appeared to develop in this case.

Vitamin B12 is involved in blood formation, neurological function, and DNA synthesis [1]. B12 deficiency causes the formation of abnormal red blood cells resulting in megaloblastic anemia, characterized by large, immature red blood cells, leading to inefficient oxygen transport and symptoms like fatigue and weakness [1,8]. B12 deficiency also impacts the nervous system, as B12 is vital for maintaining the myelin sheath. Without it, neurological functions deteriorate, leading to symptoms such as numbness, tingling, balance issues, and cognitive disturbances [1].

B12 deficiency is caused by lack of intrinsic factor (pernicious anemia, an autoimmune disease), a diet devoid of B12 (such as a vegan diet), gastrointestinal tract surgery, prolonged use of medications such as metformin or proton pump inhibitors (PPIs), and conditions like Crohn's disease. The treatment is to simply supplement with B12 in many cases [1,8]. 

MDS is a group of clonal bone marrow disorders and is characterized by ineffective hematopoiesis leading to cytopenias and potential progression to acute myeloid leukemia, with diagnostic criteria relying heavily on bone marrow morphology. While the exact cause of MDS is unknown, chemotherapy and radiotherapy are among the known risk factors [8-10].

In most cases, vitamin B12 deficiency leads to a macrocytic anemia that is typically treated early in its course with supplementation [2]. However, it is important to keep in mind that complications with any process can progress. As in this case, for example, with severe deficiency untreated for a long period of time leading to a presentation imitating MDS. An MDS look-alike secondary to vitamin B12 deficiency is becoming a more commonly published phenomenon worthy of attention [3-4,6-7]. A review of literature reveals several instances where vitamin B12 deficiency presents with hematological abnormalities mimicking MDS, including macrocytic anemia, neutropenia, and thrombocytopenia [3-4,6-7]. However, distinguishing between these conditions is crucial, as their management and prognosis differ significantly. Like many medical problems, vitamin B12 deficiency is typically minor in its overall effect on patients with early intervention. However, if it continues without treatment, even such a simple problem can result in major complications. 

The overall implications of this case are significant. Uncommon, severe presentations of disease processes do occur. Keeping a broad differential and considering the unlikely explanation for why something is happening can sometimes lead to a simple solution. The potential diagnosis of MDS is typically ominous; however, in this case, some vitamin B12 was all that was needed.

## Conclusions

Vitamin B12 deficiency can present with pancytopenia instead of solely anemia. In treating such a clinical presentation, it is important to rule out B12 deficiency before pursuing the treatment regimen of MDS by supplementing vitamin B12 and giving enough time for the marrow to resume the proliferation of all cell lines. 

## References

[1] (2024). Vitamin B12: Fact Sheet for Consumers. https://ods.od.nih.gov/factsheets/VitaminB12-HealthProfessional/.

[2] Nagao T, Hirokawa M (2017). Diagnosis and treatment of macrocytic anemias in adults. J Gen Fam Med.

[3] Kesbeh Y, Pakbaz Z (2019). Pernicious anemia: a myelodysplastic syndrome look-alike. J Community Hosp Intern Med Perspect.

[4] Kim M, Lee SE, Park J (2011). Vitamin B(12)-responsive pancytopenia mimicking myelodysplastic syndrome. Acta Haematol.

[5] Randhawa J, Ondrejka SL, Setrakian S, Taylor H (2013). What should I know before ordering a bone marrow aspiration/biopsy in patients with vitamin B12 deficiency?. BMJ Case Rep.

[6] Drabick JJ, Davis BJ, Byrd JC (2001). Concurrent pernicious anemia and myelodysplastic syndrome. Ann Hematol.

[7] Steensma DP (2012). Dysplasia has a differential diagnosis: distinguishing genuine myelodysplastic syndromes (MDS) from mimics, imitators, copycats and impostors. Curr Hematol Malig Rep.

[8] Zeidan AM, Shallis RM, Wang R, Davidoff A, Ma X (2019). Epidemiology of myelodysplastic syndromes: why characterizing the beast is a prerequisite to taming it. Blood Rev.

[9] Haferlach T (2019). The molecular pathology of myelodysplastic syndrome. Pathobiology.

[10] Hasserjian RP (2019). Myelodysplastic syndrome updated. Pathobiology.

